# The Variable Frequency Conductivity of Geopolymers during the Long Agieng Period

**DOI:** 10.3390/ma14195648

**Published:** 2021-09-28

**Authors:** Janusz Walter, Marimuthu Uthayakumar, Ponnambalam Balamurugan, Dariusz Mierzwiński

**Affiliations:** 1 Department of Materials Engineering, Faculty of Materials Engineering and Physics, Tadeusz Kosciuszko Cracow University of Technology, 37 John Paul II Avenue, 31-864 Cracow, Poland; dariusz.mierzwinski@pk.edu.pl; 2 Faculty of Mechanical Engineering, Kalasalingam Academy of Research and Education, Krishnankoil 626126, India; uthaykumar@gmail.com (M.U.); p.balamurugan@klu.ac.in (P.B.)

**Keywords:** geopolymer, electro conductivity, physical sorption analysis, porosity, XRD

## Abstract

The variable frequency conductivity was applied to characterize the process of solidification of geopolymers based on fly ash with sand additives. XRD qualitative and quantitative analysis, porosity measurements, and sorption analysis of specific surface area were performed. The conductivity was correlated with porosity and specific surface area of geopolymer concretes. Both values of conductivity, real and imaginary parts, decreased during polymerization processing time. Characteristic maximum on graphs describing susceptance vs. frequency curve was observed. The frequency of this maximum depends on time of polymerization and ageing, and can also indicate porosity of material. Low-porous geopolymer concrete shows both low-conductivity values, and susceptance maximum frequency peak occurs more in the higher frequencies than in high-porous materials.

## 1. Introduction

The development of materials with positive effects on reducing global warming and pollution generated by human activity recently became the most important proecological activity. One solution in ecology is to try to reduce greenhouse gases from the production of ordinary Portland cement (OPC) and the generation of electricity by coal-fired power plants, especially in developing countries with increased demand for infrastructure. An ecologically friendly alternative to OPC that does not involve a high-temperature reaction that generates large amounts of CO_2_ is needed [1]. Such materials can be geopolymers made of both natural and synthetic materials, which are the result of other technological processes, including fly ash. The advantage of producing these materials compared to that of OPC is that they can be produced at temperatures ranging from ambient to 80 °C by alkaline activation of a few aluminosilicate minerals [2,3,4].

Factors influencing geopolymer concrete produced with 100% fly ash as the main binder were the subject of many studies in recent years [5,6,7,8,9]. Geopolymer concrete differs significantly from Portland Cement (PC) concrete because it is easier to achieve structural integrity. PC concrete relies on the presence of calcium silicate hydrate (C-S-H) gel for matrix formation and strength development, while geopolymer concrete is subject to polycondensation of alumina and silica with an alkaline activator. The mechanism of geopolymerization involves three main steps: dissolving the oxides of alumina and silicon in the alkaline activator; transport of dissolved grades of alumina and silica; and polycondensation with formation of an aluminosilicate gel as the main reaction product [10]. In the activation process, hydroxyl anions act as a catalyst for the reaction, while alkali metal cations act as structure-forming elements. The structure of the aluminosilicate gel contains Si^4+^ and Al^3+^ randomly distributed along the polymer chains that are cross-linked while providing spaces of sufficient size to accommodate the charge-balancing hydrated alkaline cations.

The main challenge faced by the construction industry when adopting geopolymeric material is the variability of fly ash from various sources and the impact it may have on the compressive strength of the produced geopolymers [11,12]. This variation in composition may result in obtaining geopolymeric concretes with microstructures. So far, many parameters influencing the compressive strength of geopolymers were identified, such as particle size distribution, fly ash specific surface, SiO_2_/Al_2_O_3_ and Na_2_O/Al_2_O_3_ ratio, amorphous content, CaO content, workability, and liquid/solid and Na_2_SiO_3_/NaOH ratios [13,14,15,16].

Considering that the content of Al_2_O_3_ and SiO_2_ and their dissolution are key factors in the formation of the aluminosilicate gel and the produced geopolymer matrix, it is important to determine after what time the polycondensation process ends and how it affects the microstructure of geopolymers.

Although there are now more and more indications that geopolymers are less environmentally friendly than previously claimed, given the production of the alkaline activator and factors such as energy-intensive processes in which some geopolymer precursors are produced [17,18]; these factors were often overlooked or ignored by earlier geopolymer advocates. Nevertheless, until now, geopolymers were of major interest as an alternative to OPC, although they have many other technologically advanced applications [19,20,21].

When analyzing the physicochemical properties of geopolymers, special attention should be paid to the ion exchange of these materials, especially in an environment with high humidity or even in contact with watercourses [22,23,24].

Due to the geopolymer’s ability to adsorb and immobilize hazardous material in its structure, the environmentally friendly interaction of geopolymers makes them potentially important materials for mitigating climate change issues [25,26].

This article presents important aspects related to the chemical processes occurring during the aging of geopolymers where the constant value was the composition of the dry mass used in the production of alkaline activated materials. The variable value was the use of three different molar concentrations of NaOH in the alkaline solution. The influence of the binding time on the ion exchange and the end of the polycondensation process was demonstrated using variable frequency conductivity measurements taken in a specially designed electrode setup across a long ageing time, while geopolymers were stored in ambient conditions. Typically, concretes conductivity is measured using two-point uniaxial method or Wenner four-point probe method [27,28,29]. The second one is widely accepted but is sensitive to the surface condition. Proposed two-electrode method buried in geopolymer concrete does not have this disadvantage and is sensitive enough to measure concrete after 3 years of ageing what was shown. This method provides information about progress of solidification processes in time and the influence of the geopolymer composition on it. The simultaneous influence of the porosity changing with the molar concentration of the alkaline solution were also demonstrated.

Unfortunately, some of the studies reported so far did not pay enough attention to these requirements, suggesting a need for a more rigorous approach in future studies.

## 2. Materials and Methods

### 2.1. Materials and Sample Preparation

Mixtures of fly ash from the Skawina CHP plant (Skawina, Poland) and construction sand in a ratio of 1:1 were activated by addition of different concentrated NaOH water solution (8 M, 10 M and 12 M), each modified by addition of sodium silicate R-145 aqueous solution with a 2.5 molar module.

The oxide composition of the fly ash determined by the XRF method is shown in Table 1.

**Figure 1:**
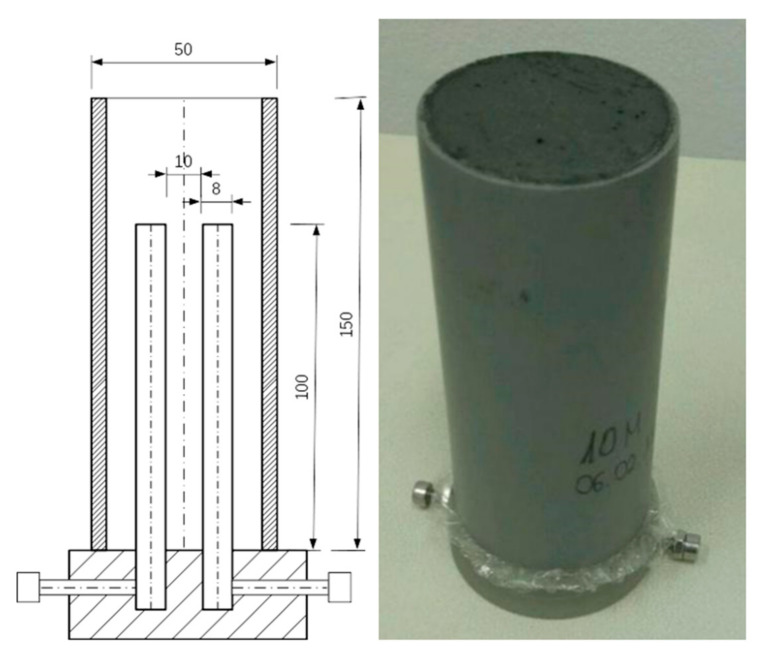
Schema of form containing electrodes and picture of geopolymer in form.

Components were initially dry mixed for 10 min and in a next step NaOH activator was added. Wet mixing was realized over another 10 min. Fresh mixtures were poured into specially prepared cylindrical forms containing two electrodes made of stainless steel and were vibrated. The vibrating table was used to release any residual air bubbles. Forms were prepared using 150 mm long PCV tube diameter of 50 mm. Distance between cylindrical steel electrodes was set to 10 mm and kept along tube axis by design for the purpose of holding the electrodes. Design of the form is shown in Figure 1.

Forms containing electrodes kept in holders were covered with thermal-resistant plastic foil to prevent water evaporation were cured in oven at 75 °C for 24 h after casting. Afterwards, geopolymers were cooled in ambient conditions. Composition of geopolymers is shown in Table 2. Description G8M means that 8 M solution of NaOH was used, and consequently, G10M means 10 M and G12M is 12 M of solution of NaOH was applied as activator.

### 2.2. Conductivity Measurements

Three hours after removing forms with geopolymers from the oven, the concretes obtained room temperature. Using short electrical cables, stainless steel electrodes were connected to the Zahner IM6e workstation to measure conductivity of solidified materials. The workstation was set in two electrodes mode without any additional electrode polarization. The variable frequency amplitude of 50 mV was applied between electrodes. Frequency of the current was changed during the measurement in the range of 1 Hz to 10 kHz. Obtained geopolymer concretes were stored without removing of PCV tubes and electrodes in a desiccator in an ambient temperature to prevent water evaporation from materials and to keep stable humidity around them.

Measurements were performed periodically across three years of storage in these conditions. The occurrence of so-called efflorescence, which was previously investigated [24,30], was not observed during whole period of storage. Materials were removed from forms after 3 years and immersed in distilled water to transfer soluble salts out of the geopolymer. Water was periodically exchanged to accelerate diffusion of soluble ions into solution. Immersion of materials in the water process was finished after 30 days. Afterwards, conductivity of surface-dried and soaked concretes were checked. The next step was to remove all water from geopolymers by heating them in the drier at a temperature of 70 °C for 48 h, and to test again for conductivity of dry concrete.

### 2.3. XRD Methods

An Aeris, PANalytical X-ray diffractometer was used for XRD analysis. The measurement parameters are presented in the Table 3.

### 2.4. Physical Sorption Analysis

The research was carried out using the Quantachrome Autosorb iQ-MP physical sorption analyzer.

Nitrogen adsorption and desorption isotherms were obtained in the range of relative pressures (P/P_0_): 1·10^−6^ ÷ 0.95. Measurement cells with an outer diameter of ø 12 mm (wall thickness 1 mm) were used. A filling rod was used during the measurement.

The degassing process took place in several stages and is presented in the Table 4.

Each time after the degassing and measurement process, the properties of the tested samples were analyzed with the Quantachrome ASiQwin program. The specific surface area was determined by the Brunauer–Emmett–Teller multipoint method (BET) in the range of relative pressures P/P_0_ from 0.05 to 0.30 and by the single point BET method at P/P_0_ of 0.1. In addition, the volume and diameter of the pores were determined using the method of Barret, Joyner, Halenda (BJH). The microporosity of the samples was determined using the Dubinin–Raduszkiewicz (DR) method.

### 2.5. Porosity

Porosity testing of geopolymers was performed after three years of ageing with the use of a Quantachrome Poremaster 33 mercury porosimeter, within a pressure range of 1 to 400 MPa. Test results were expressed in the volumetric content of pores, and distribution curves of pore sizes were within a range of 0.0035–1.075 μm.

## 3. Results

### 3.1. Results of Conductivity Measurements

Geopolymer concretes obtained were solid materials after 24 h of heating in the oven. The first measurement of the conductivity was performed after 52 h passed and it stabilized at room-level temperature; stabilized conductive properties are not dependent on temperature.

#### 3.1.1. G8M

**Figure 2:**
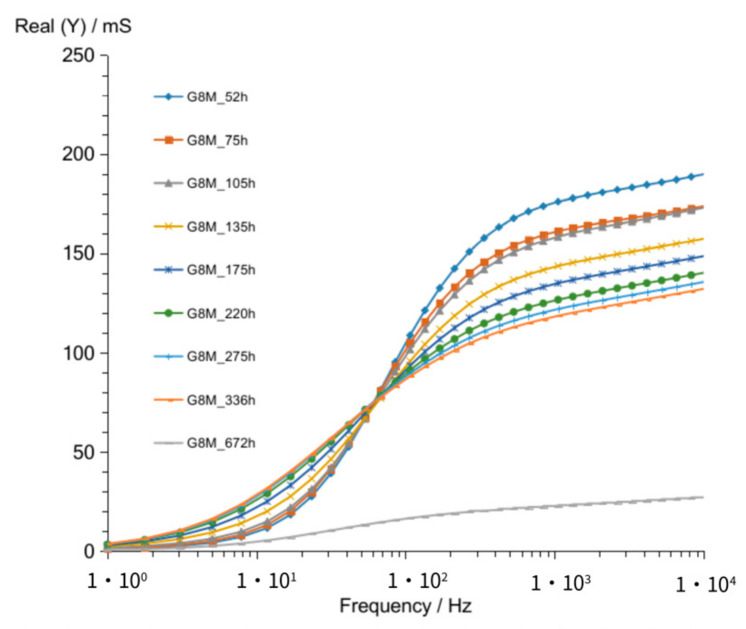
Admittance of G8M geopolymer concrete taken after 52 up to 670 h from starting polymerization.

The admittance curves (Figure 2) describe real component of conductivity versus current frequency. Curves showed in Figure 2 represent measurements obtained after 52 h up to 336 h passed from starting point of formation of geopolymer concretes G8M. Those curves crossed at about 68 Hz. Increasing the time of storing in ambient temperature at constant humidity in the frequencies over 68 Hz resulted in shifting admittance curve to the lower conductivity with time. Admittance values range from about 176 mS to 118 mS at 1 kHz frequency. Lower values correspond to longer time of storage. The measurement taken after 672 h was included in graph for easier comparison of curve shape obtained after longer time.

**Figure 3:**
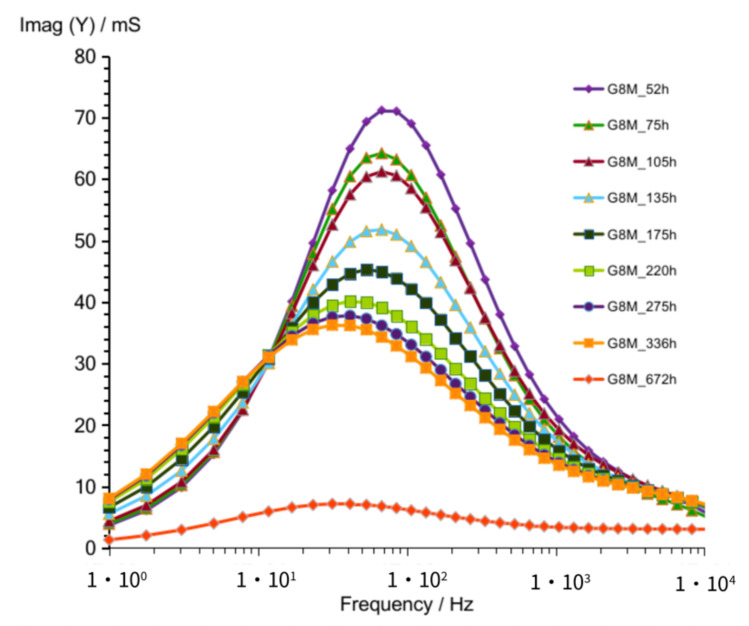
Susceptance of G8M geopolymer concrete taken after 52 do 670 h from starting polymerization.

Parallel obtained curves of susceptance (Imag (Y)) for these measurements were shown separately in Figure 3 for better clarity of graphs. The graph shows maximum in all curves, which is obtained as the frequency changes from about 70 to 30 Hz while the storage time increases from 52 to 336 h. The decreasing values of those maximums are seen in graph while time increases.

The lowest curves (Figure 2 and Figure 3) show result obtained after 672 h passed. A significant difference is shown between curve obtained after 672 h compared to that of other curves achieved after shorter periods of time.

**Figure 4:**
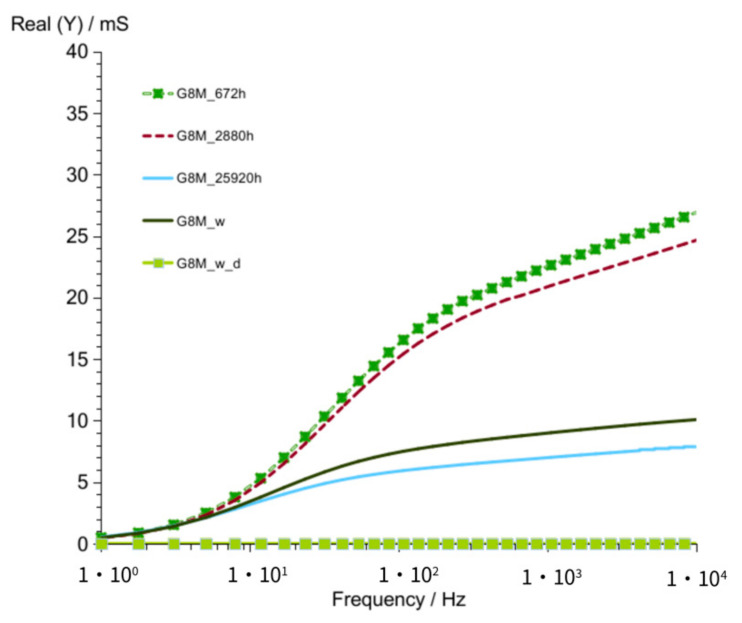
Admittance of G8M geopolymer concrete taken after 672 to 25,900 h from starting polymerization after removing conductive ions (G8M_w) and drying concrete (G8M_w_d).

**Figure 5:**
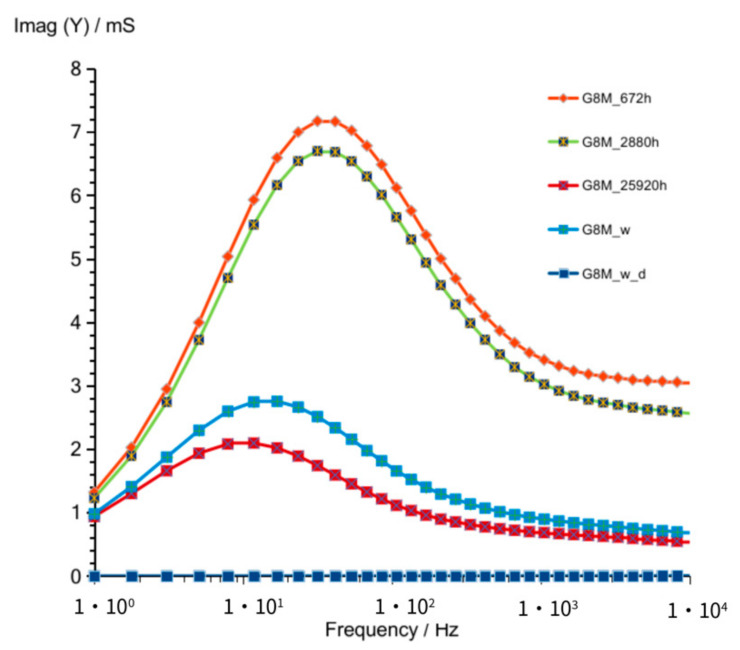
Susceptance of G8M geopolymer concrete taken after 672 to 25,920 h from starting polymerization after removing conductive ions (G8M_w) and drying concrete (G8M_w_d).

Results for longer time of storage achieved for the concrete G8M was shown in Figure 4 and Figure 5. An admittance was shown for 672 h (28 days), 2880 h (4 months), 25,900 h (36 months), and additionally, in the end for the same geopolymer concrete after its rinsing in water to remove soluble substances, which could influence conductivity (G8M_w). The curve of the geopolymer dried in the next step was marked as G8M_w_d.

Changes of susceptance versus frequency (Figure 5) observed for measurements taken after 672 and 2880 h showed only small shift to the lower values after longer time looking on vertical axe values. The same occurs on Figure 4 for those measurements—slightly lower values of admittance observed after longer time. Concretes were stored up to 25,920 h (3 years) in desiccator and tested again. The admittance taken after 3 years showed much lower values in higher frequencies compared to that of values obtained after 4 months. The same direction of changes can be seen in susceptance curves. That shows progressive changes in concrete while the time passed. It is the result of decreasing amount of well-conductive substances as water and conductive ions were added to the activator. If we take into account that concretes were stored in desiccator, a decrease in conductivity should result, mainly by slow progressive reactions which lead to a smaller number of ions taking part in charge transfer. Curves shown in Figure 4 and Figure 5 describe G8M_w being obtained after removing conductive ions (not considering water) possibly contained in the concrete by immersing geopolymer in water, as described in article [24]. Curves on both figures show little better conductivity compared to that of curves G8M_25920 h. This is a result of water presence that was left in pores after immersing.

The last measurements included in those figures were achieved after removing water from concrete. Curves described in figures as G8M_w_d show both values of conductivity (Real (Y and Imag (Y)) equal to 0 mS. We found that concrete after drying is an insulator.

**Figure 6:**
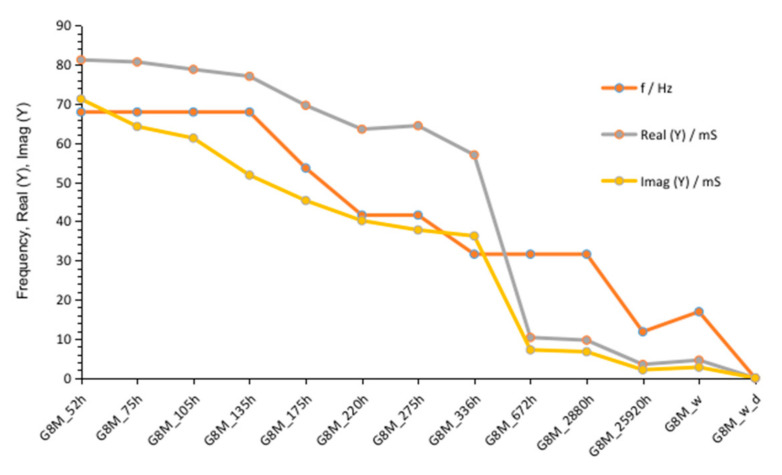
Changes of susceptance maximum and corresponding values: admittance and frequencies, depending on time passed from polymerization beginning; after removing conductive ions (G8M_w); after drying concrete (G8M_w_d).

The susceptance values included in Figure 3 and Figure 5 showed characteristic maximum values visible on graphs as peaks. We found slow shifting of them into lower frequency depending on the time. These changes were summarized and shown in Figure 6 and Table 5. Values of maximum peak of Imag (Y) and corresponding values Real (Y) and frequency indicates peaks were also included on graph. Both values of conductivity decrease while time changes to 336 h. Imag (Y) moves from about 71 mS to about 36 mS and Real (Y) from about 68 to 32 mS. Frequency of admittance peak changed from 68 to 32 Hz. The next measurement taken after 672 h showed much smaller conductivity equal about 10 mS on both curves at the same frequency as previous measurement. This probably indicates the end of significant changes in geopolymer concrete. The further measurements showed smaller decreasing of conductivity and peaks occurs in lower frequencies.

#### 3.1.2. G10M

**Figure 7:**
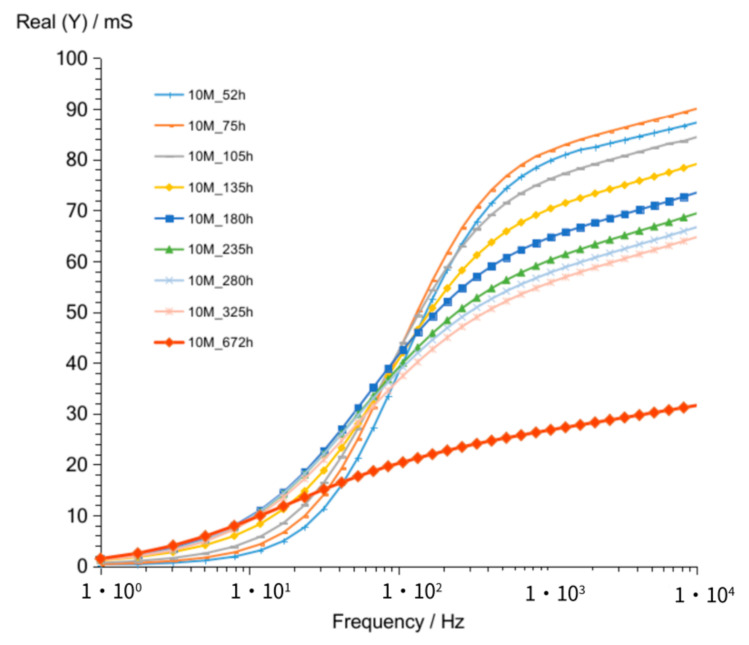
Admittance of G10M geopolymer concrete taken after 52 to 672 h from starting polymerization.

**Figure 8:**
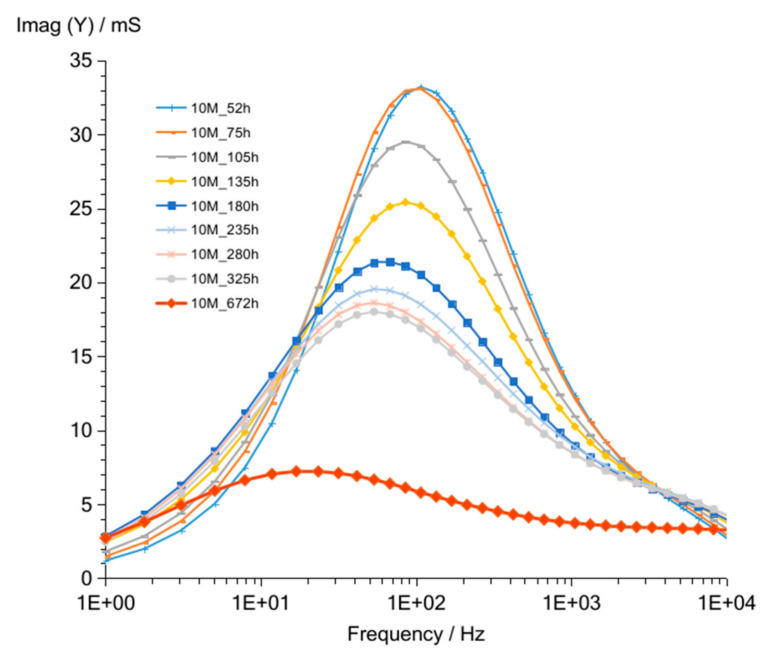
Susceptance of G10M geopolymer concrete taken after 52 to 672 h from starting polymerization.

The admittance curves (Figure 7) obtained for G10M measurements look similar to G8M curves. Curves obtained after 52 up to 336 h crossed at about 90 Hz. The crossing frequency is higher compared to that of G8M, and admittance values ranging from about 82 to 56 mS at 1 kHz frequency are shifted to smaller values while time increases (Table 5). The measurement taken after 672 h showed similar curve shape to G8M but slightly higher values (about 5 to 6 mS) at frequencies over 90 Hz. Figure 8 shows changes in susceptance curves Imag (Y) versus frequency. The maximum in all curves changing form about 100 to 50 Hz while the storage time passed from 52 h to 336 h. The lowering value of admittance for those maximums are seen in graph also while time is increasing, as was found in G8M concrete.

**Figure 9:**
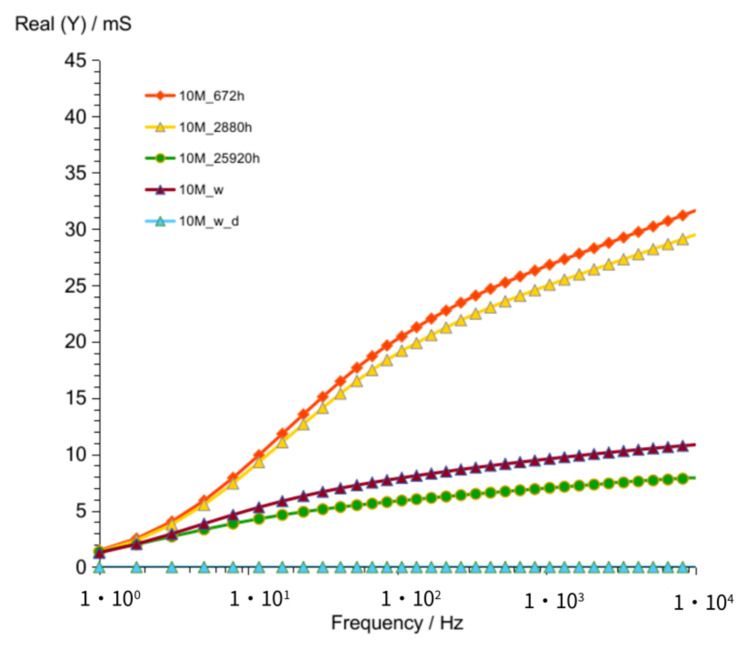
Admittance of G10M geopolymer concrete taken after 672 to 25,920 h from starting polymerization after removing conductive ions (G10M_w) and drying concrete (G10M_w_d).

**Figure 10:**
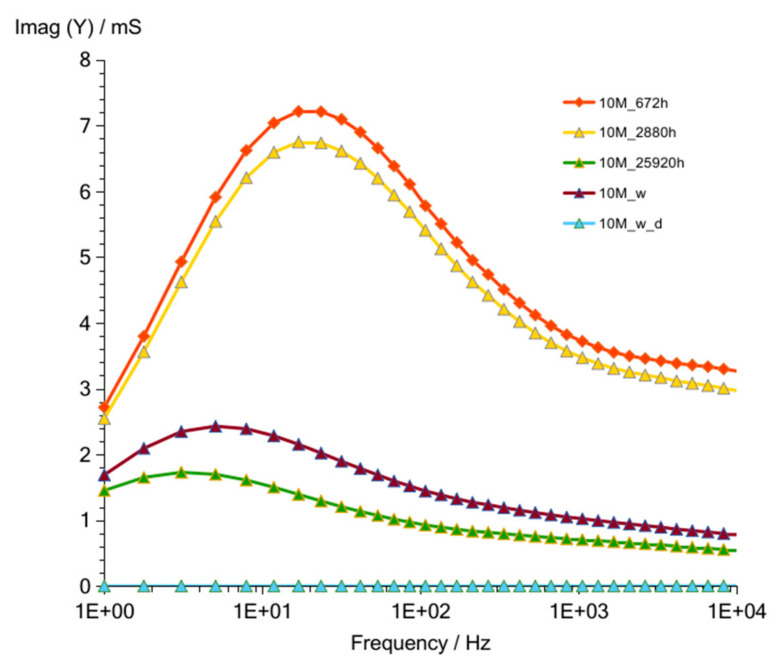
Susceptance of G10M geopolymer concrete taken after 672 to 25,920 h from starting polymerization after removing conductive ions (G10M_w) and drying concrete (G10M_w_d).

**Figure 11:**
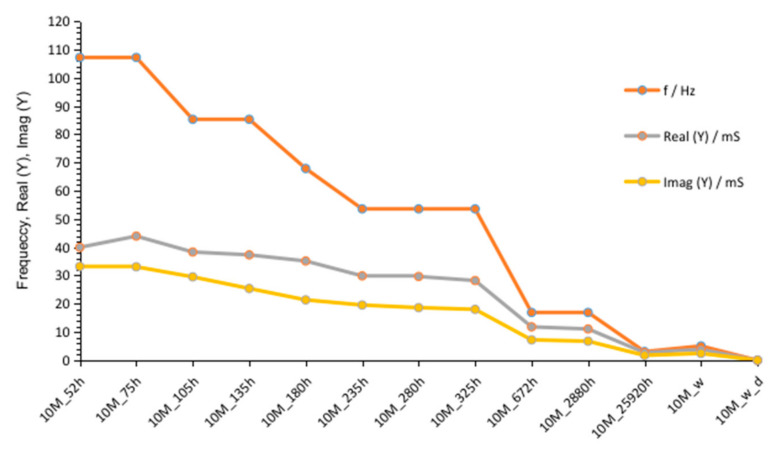
Changes of frequencies, admittance, and susceptance depending on time passed from starting polymerization after removing conductive ions (G10M_w) and drying concrete (G10M_w_d).

Results obtained after 672 h (Figure 9 and Figure 10) showed, as in previous measurement (G8M_672 h), much lower values compared to that of other curves achieved after shorter periods of time. Summarized changes in curves are shown in Figure 11. Values of maximum peak of Imag (Y) moves from 33 to 18 mS while time of storage goes from 52 to 325 h and corresponding Real (Y) values moves from about 44 to 28 mS. During this period frequency of admittance peak changed from 100 to 50 Hz. The next measurement taken after 672 h showed much smaller conductivity equal about 10 mS on both curves at the same frequency as previous measurement (G8M). The further measurements showed decreases in conductivity and peaks occurring at lower frequencies.

#### 3.1.3. G12M

**Figure 12:**
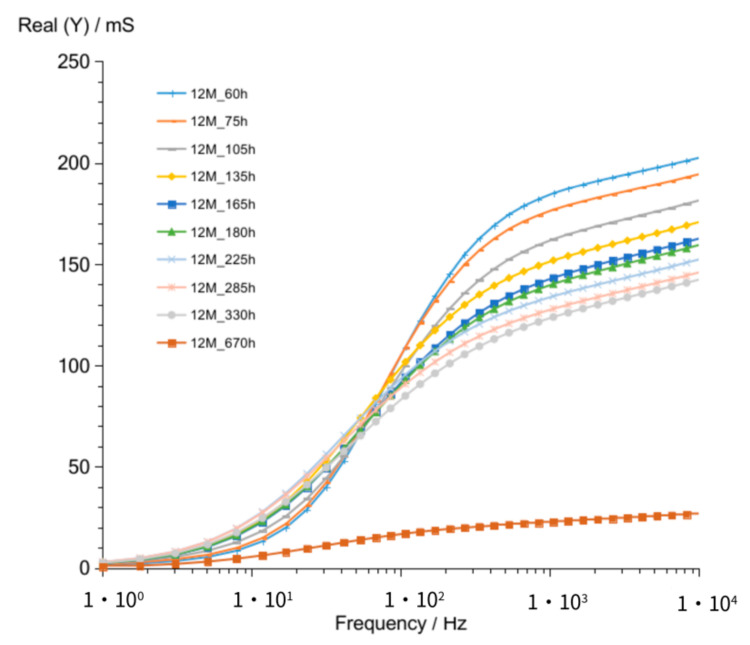
Admittance of G12M geopolymer concrete taken after 60 to 670 h from starting polymerization.

**Figure 13:**
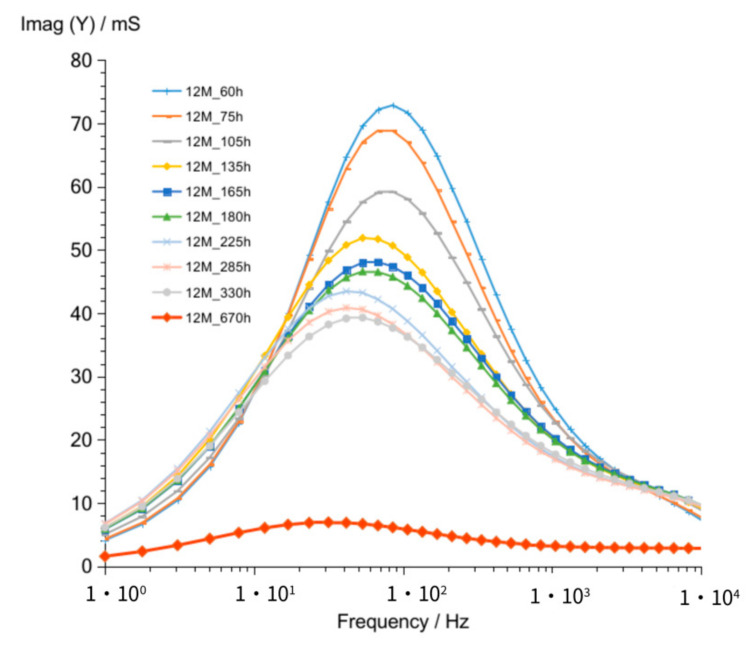
Susceptance of G12M geopolymer concrete taken after 60 to 670 h from starting polymerization.

The shape of admittance curves (Figure 12) obtained for G12M geopolymer looks similar to that of G8M and G10M curves. Curves obtained after 60 up to 330 h crossed at about 68 Hz. The crossing frequency is equal to G8M and lower compared to that of G10M. The admittance values (Figure 12) range from about 185 to 123 mS at 1 kHz frequency are about two times higher than that obtained for G10M but are comparable to that of G8M. Figure 13 shows changes in susceptance curves Imag (Y) versus frequency. The maximum in all curves changing form about 90 to 50 Hz, while the storage time passed from 60 to 330 h. The lowering value of admittance for those maximums are seen in graph also while time is increasing.

**Figure 14:**
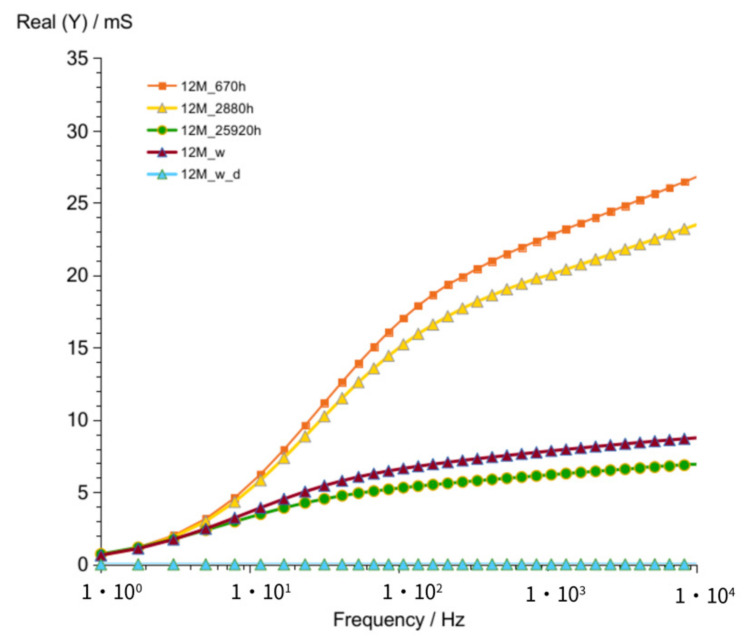
Admittance of G12M geopolymer concrete taken after 670 to 25,920 h from starting polymerization after removing conductive ions (G12M_w) and drying concrete (G12M_w_d).

**Figure 15:**
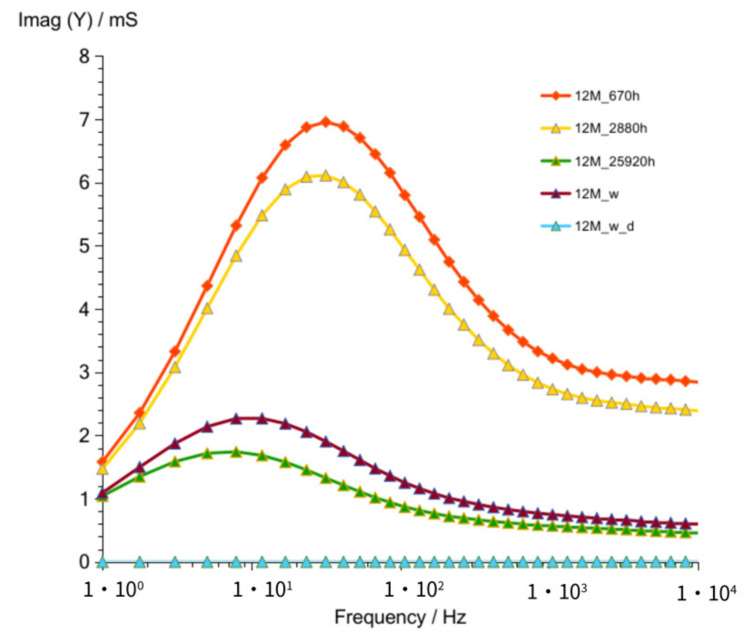
Susceptance of G12M geopolymer concrete taken after 670 to 25,920 h from starting polymerization after removing conductive ions (G12M_w) and drying concrete (G12M_w_d).

Results obtained after 670 h (Figure 14 and Figure 15) showed as in previously measured geopolymers (G8M and G10M) much lower values compared to that of other curves achieved after shorter periods of time.

**Figure 16:**
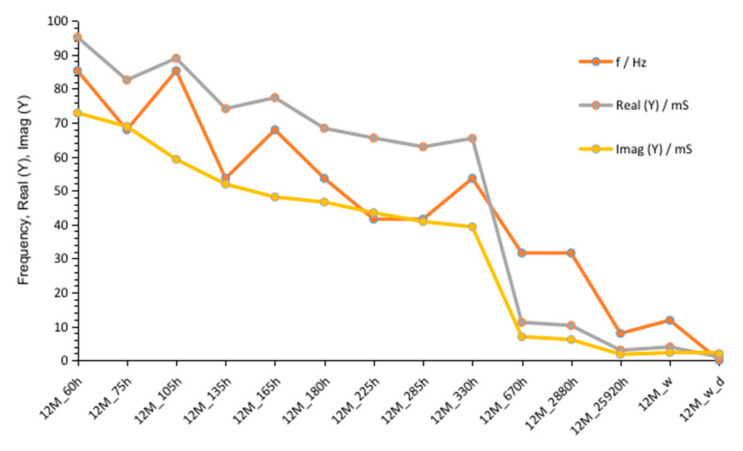
Changes of frequencies, admittance, and susceptance depending on time passed from starting polymerization after removing conductive ions (G12M_w) and drying concrete (G12M_w_d).

Summarized changes in curves are shown in Figure 16. Values of maximum peak of Imag (Y) moves from 73 to 39 mS, while time of storage goes from 60 to 330 h and corresponding Real (Y) values moves from about 95 to 65 mS. During this period, frequency of admittance peak changed from 90 to 50 Hz. The next measurement taken after 670 h showed much smaller conductivity, equal to about 10 mS on both curves. The further measurements showed smaller decreases in conductivity but peaks occurred at lower frequencies.

Summary of Conductivity Measurements.

**Table 5:** Characteristics of geopolymer values obtained from listed figures—usable for comparison.

	Value Describing	G8M	G10M	G12M
Figure 2 Figure 7 Figure 12	crossing curves frequency—Real (Y)	68 Hz	90 Hz	68 Hz
	values of Real (Y) at 1 kHz (at time about 60 to 320 h)	176–118 mS	82–56 mS	185–123 mS
	values of Imag (Y) at 1 kHz (at time about 60 to 320 h)	21–13.5 mS	12–8.3 mS	24.7–17.7 mS
Figure 3 Figure 8 Figure 13	frequency of susceptance maximum peak—Imag (Y) (at time about 60 to 320 h)	70–30 Hz	100–50 Hz	90–50 Hz
Figure 6 Figure 11 Figure 16	values of Real (Y) at frequency of susceptance maximum peak (at time about 60 to 320 h)	68–32 mS	44–28 mS	95–65 mS
Figure 6 Figure 11 Figure 16	values of Imag (Y) at frequency of susceptance maximum peak (at time about 60 to 320 h)	71–36 mS	33–18 mS	73–39 mS

### 3.2. Results of XRD

The analysis of the obtained diffraction spectra was performed with the HighScore Plus software.

**Figure 17:**
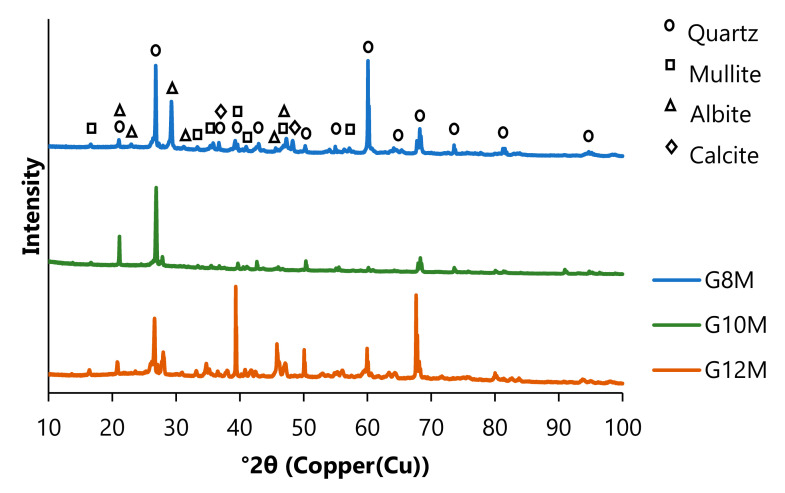
XRD of geopolymers after three years of ageing, resoaking in distilled water, and drying.

Diffraction patterns obtained for investigated geopolymers (Figure 17) showed important differences between concretes. G10M consists of the biggest content of quartz and smallest values of mullite, albite, and calcite (Table 6) compared to that of G8M and G12M. Spectrum of G12M geopolymer consists of more quartz and less of other compounds than that of the G8M.

### 3.3. Results of Porosity

**Figure 18:**
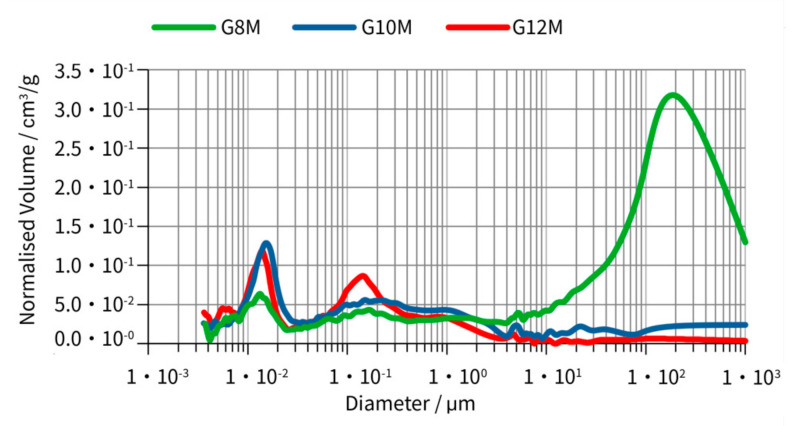
Comparison of porosity distribution for geopolymer samples after three years of ageing, resoaking in distilled water, and drying.

Porosity checked on mercury porosimeter was shown in Figure 18.

Total porosity decreases with increasing of amount of activator (Table 7). The smallest porosity was achieved in the G12M geopolymer. The interparticle porosity of G8M is about 30 times bigger than that of the G12M. The largest volume of pores was found in G8M concrete. This was caused by a large number of pores about size of 200 μm (Figure 18). The intraparticle porosity, which generally describes smaller pores, obtained biggest values with G10M and smallest with G8M (Table 7). The geopolymer G12M mainly consists of a smaller number of pores than G10M, but at pore size of about 0.15 μm, normalized volume G12M is about 70% bigger than that of G10M (Figure 18).

### 3.4. Results of Physical Sorption Analysis

Result of sorption analysis showed the biggest specific surface area for G10M and calculated an average pore diameter of about 11 nm. Concretes G8M and G12M obtained lower specific surface areas (Table 8).

## 4. Discussion

There are some investigations reported in literature focused on measuring resistivity of mortars and concretes [31,32,33,34,35,36,37]. The aim of these works was to increase conductivity, which is needed for health monitoring of geopolymer structure. Most of the works were based on uniaxial two-point AC current method.

The mentioned methods should be accurate in case of repeatable connection of electrodes attached externally to the geopolymer. Setup designed for our investigations has no disadvantage and should have the same resistivity of electrode-geopolymer connection. All measurements taken for the same sample should characterize the same charge transfer resistance if we assume that there are no reactions on the electrodes. Conductivity of tested samples in proposed setup depends on conductivity of geopolymer concrete in that case.

We found our proposed conductivity checking method can be used for characterization of geopolymer solidification process. Results of conductivity investigation measured using alternating current showed in all tested geopolymer concretes characteristic maximum peak on susceptance curves in the range of about 30 to 100 Hz. The value of maximum susceptance peak separates the dominant conductivity type. The capacitance conductivity dominates in frequencies lower than frequency of maximum peak, but the resistance type of conductivity also took place and continuously decreased while frequency went down. The opposite situation occurs in frequencies higher than the maximum peak frequency. The conductivity type changes from the capacitance conductivity to the resistance conductivity. The highest frequencies represent mainly the resistance conductivity, but we still recorded in these frequencies susceptance values higher than zero and cannot say that we measured exact value of electrical conductivity. The lower maximum frequencies corresponded in general with longer time of geopolymer storage in ambient conditions. That means, conductivity becomes more resistive type. Values of admittance and susceptance obtained at frequency of peak concurring also decreased with time passing for each geopolymer curve’s downward shift. Materials became less conductive in general. Curves of admittance changes from ascending to descending at maximum of susceptance peak. Continues changes of conductivity were observed up to about 320 h (2 weeks). The next measurements taken after about 670 h showed that changes in concrete almost stopped—conductivity was not changed. The extension of time to 2,880 h result in very similar to 670 h values (Figure 5, Figure 10, and Figure 15). We conclude process of changes in geopolymers stopped. Measurements taken after 3 years (25,920 h) showed that measurements can be also realized and small but not significant changes can be recorded. The same changes are observed in all geopolymers: G8M, G10M, and G12M. The lower conductivity (both values—Real (Y) and Imag (Y)), was found for concrete G10M. Table 5 shows generally two times lower values of admittance and susceptance at 1 kHz compared to that of G8M and G12M. The same was found at susceptance maximum peak. The crossing curves frequency obtained the highest value equal to 90 Hz for G10M. This frequency in general has the similar value as frequency of susceptance maximum peak. It was found that changes in G8M concrete goes slower than for the other two concretes. Curves crossed in one point looking at curves G8M_52h to G8M_336h (Figure 2). As it was written before curves moves down in the graph when the time is passing, we found that corresponding curves for G10M and G12M move down faster and did not cross at exactly one point because of bigger amount of alkaline activator. Both of the other geopolymers, G8M and G12M, showed much lower crossing frequency—68 Hz. The difference between values of conductivity in G8M and G12M does not offer clear information about the difference between geopolymers, but the susceptance maximum peak of G12M is observed in higher range of frequency (similar to G10M) compared to that of G8M (Table 5). Dried geopolymers: G8M__w_d_, G10M__w_d_ and G12M__w_d_ are not conductive materials. We found that a small number of conductive ions is necessary to get charge transfer in pores. The source of conductive ions in geopolymers is the ambient atmosphere humidity. We found G10M is the best geopolymer among the tested. It characterizes the smallest values of conductivity (Table 5) and the highest susceptance maximum peak frequency. That means the conductivity is more capacitive type and concrete is less conductive. Results of porosity testing corresponds to conductivity values. G10M geopolymer has biggest intraparticle porosity, small interparticle porosity (3.19%)—Table 7. This should result in decreasing of conductivity because of higher charge transfer resistance in small or intraparticle pores what was found. Specific surface area (Table 8) obtained by sorbtion analysis corresponds to conductivity in the same way as intraparticle porosity. The big specific surface area and small size of pore diameter worsened conductivity.

## 5. Conclusions

We compared fly ash geopolymers formation using the proposed variable frequency conductivity method and found that the characteristic maximum susceptance peak occurs at higher frequencies in low-porous geopolymers after the same duration of ageing. Frequency of this peak moved to the lower values as time of polymerization passed. The lower geopolymer concrete porosity resulted in smaller values of admittance and susceptance compared to that of other geopolymers. Conductivity gradually decreases in time of solidification. The process of geopolymer formation stopped after four weeks. The main reactions already took place, and the number of ions that can participate in conduction decreased. Drops in both conductivity values were indicative of that. The longer time of ageing resulted in much smaller changes but showed a continuously decreasing number of conductive ions. Dried geopolymer is not conductive, so observed small conductivity after a very long period can be caused mainly by ions whose source is humidity of ambient atmosphere.

Therefore, the presented measurement method allows to determine the end of the polycondensation process in geopolymers, and to analyze the phenomena occurring during this process. The method can also be used for geopolymer comparison, according to what was confirmed by XRD and porosity tests of investigated materials.

## Figures and Tables

**Table 1:** Fly ash oxide composition.

Oxide Composition [wt%]								
SiO_2_	Al_2_O_3_	Fe_2_O_3_	K_2_O	CaO	MgO	TiO_2_	P_2_O_2_	Na_2_O
55.89	23.49	5.92	3.55	2.72	2.61	1.09	0.82	0.59

**Table 2:** Geopolymers composition.

Sample No.	Aqueous NaOH [mL] + Aqueous Sodium Silicate (Water Glass) [mL]	Fly Ash [g]	Sand [g]
G8M	120 + 120	1000	1000
G10M	120 + 120	1000	1000
G12M	120 + 120	1000	1000

**Table 3:** X-ray diffraction (XRD) analyzer settings.

Parameters	Components
Angular range: 9.999 ÷ 100°2Ѳ	Nickel filter on the lamp
Measuring step: 0.0027166°2Ѳ	13 mm mask
Counting time: 340,425 s	Slot 1°
Total measurement time: 13:02:32	Blade in low position

**Table 4:** Sample degassing process.

Temperature [°C]	Heating Speed [°/min]	Time of Degassing [min]
80	2	30
120	2	30
350	5	300

**Table 6:** Qualitative and quantitative analysis of identified phases in geopolymers after three years of ageing, resoaking in distilled water, and drying.

Sample No.	Percentage [%]			
	Quartz SiO_2_ ICDD PDF 01-070-3755	Mullite Al_6_Si_2_O_13_ ICDD PDF 00-015-0776	Albite NaAlSi_3_O_8_ ICDD PDF 01-080-3255	Calcite CaCO_3_ ICDD PDF 00-003-0596
G8M	32.3	24.5	42.2	1.1
G10M	54.8	18.3	26.8	0.1
G12M	37.0	24.2	38.0	0.7

**Table 7:** Results of porosity test of samples after three years of ageing and resoaking in distilled water and drying.

Sample No.	Total Porosity [%]	Interparticle Porosity [%]	Intraparticle Porosity [%]	Mercury Intrusion Porosity [%]	Pore Tortuosity	Solid Compressibility [m/N]
G8M	29.8338	23.4095	6.4243	29.8338	1.8929	1.2476 × 10^−10^
G10M	12.3247	3.1909	9.1338	12.3247	2.0907	6.9597 × 10^−11^
G12M	9.0900	0.6878	8.4022	9.0900	2.1273	9.6314 × 10^−11^

**Table 8:** Results from geopolymer physical sorption analysis after three years of ageing and resoaking in distilled water and drying.

Sample No.	Specific Surface Area [m^2^/g]		Pore Volume [cm^3^/g]			Pore Size [nm]		
	BET One-Point Method	BET Multi-Point Method	Total Pore Volume at a Single Point (P/P_0_ = 0.95)	BJH Pore Volume	DR Pore Volume	Average Pore Diameter	BJH Average Pore Diameter	DR Average Pore Diameter
G8M	8.485	9.531	0.02640	0.02507	0.00325	11.08	17.429	1.636
G10M	10.541	11.628	0.03204	0.03077	0.00409	11.02	12.325	1.676
G12M	8.385	9.464	0.02382	0.02382	0.02289	10.07	12.368	1.763

## Data Availability

Department of Materials Engineering, Faculty of Material Engineering and Physics, Cracow University of Technology, Jana Pawła II 37, 31-864 Cracow, Poland.
